# The cellular function and molecular mechanism of formaldehyde in cardiovascular disease and heart development

**DOI:** 10.1111/jcmm.16602

**Published:** 2021-05-10

**Authors:** Ying Zhang, Yanyan Yang, Xiangqin He, Panyu Yang, Tingyu Zong, Pin Sun, Rui‐cong Sun, Tao Yu, Zhirong Jiang

**Affiliations:** ^1^ Department of Cardiac Ultrasound The Affiliated Hospital of Qingdao University Qingdao China; ^2^ Department of Immunology Basic Medicine School Qingdao University Qingdao China; ^3^ Institute for Translational Medicine The Affiliated Hospital of Qingdao University Qingdao China

**Keywords:** atherosclerosis, cardiovascular disease, formaldehyde, heart development, semicarbazide‐sensitive amine oxidase

## Abstract

As a common air pollutant, formaldehyde is widely present in nature, industrial production and consumer products. Endogenous formaldehyde is mainly produced through the oxidative deamination of methylamine catalysed by semicarbazide‐sensitive amine oxidase (SSAO) and is ubiquitous in human body fluids, tissues and cells. Vascular endothelial cells and smooth muscle cells are rich in this formaldehyde‐producing enzyme and are easily damaged owing to consequent cytotoxicity. Consistent with this, increasing evidence suggests that the cardiovascular system and stages of heart development are also susceptible to the harmful effects of formaldehyde. Exposure to formaldehyde from different sources can induce heart disease such as arrhythmia, myocardial infarction (MI), heart failure (HF) and atherosclerosis (AS). In particular, long‐term exposure to high concentrations of formaldehyde in pregnant women is more likely to affect embryonic development and cause heart malformations than long‐term exposure to low concentrations of formaldehyde. Specifically, the ability of mouse embryos to effect formaldehyde clearance is far lower than that of the rat embryos, more readily allowing its accumulation. Formaldehyde may also exert toxic effects on heart development by inducing oxidative stress and cardiomyocyte apoptosis. This review focuses on the current progress in understanding the influence and underlying mechanisms of formaldehyde on cardiovascular disease and heart development.

## 1 INTRODUCTION

Formaldehyde, a colourless gas with a strong pungent door, possesses the simplest chemical structure among aldehydes. In addition to its existence within nature, formaldehyde is also widely utilized in industrial production and consumer products or generated as a by‐product of automobile exhaust, fire and cigarette smoke. As a recognized chemical preservative and tissue fixator, formaldehyde is also frequently applied to help evaluate the histological and pathological anatomy of cells and tissues.^1^, ^2^, ^3^ Moreover, formaldehyde is ubiquitous in human body fluids and tissues. The concentration of formaldehyde in cells can reach 0.2‐0.5 mM, and the formaldehyde content in the blood of healthy individuals is 0.05‐0.1 mM.^3^, ^4^ Although formaldehyde in the body is derived from both endogenous and exogenous pathways, low physiological levels can be maintained in human blood and tissues by physiological and metabolic clearance mechanisms.^5^

Nevertheless, exposure to high‐dose formaldehyde increases the risk of poisoning and prolonged exposure also carries the potential risk of causing cancers. Accordingly, the International Agency for Research on Cancer (IARC) classifies formaldehyde as a known human carcinogen.^2^ Moreover, owing to its toxicity, formaldehyde also participates in the onset of neurodegenerative diseases such as ageing and Alzheimer's disease^6^ and can cause cardiovascular diseases (CVD) such as atherosclerosis (AS),^7^ arrhythmia^8^ and stroke^9^ as well. In addition, the exposure of pregnant women to formaldehyde mainly through pre‐pregnancy or during pregnancy not only damages the body of the pregnant woman but also endangers the growth and development of the foetus. In particular, studies have revealed that exposure to formaldehyde during the first trimester of pregnancy can have deleterious effects on the developing foetus, increasing the likelihood of malformations of the foetal heart and defects leading to spontaneous abortion.^10^, ^11^ Notably, according to research by the Danish National Birth Cohort (DNBC), congenital abnormalities in the general population may be associated with non‐occupational exposure to paint and smoke during the first trimester.^12^ However, in most cases, the harmful effects of formaldehyde will continue throughout pregnancy and lead to cumulative effects such as congenital abnormalities (eg congenital heart disease, limb defects, gastrointestinal obstruction and cleft lip and/or palate), foetal death and low birth weight.^13^

In this review, we describe the mechanism of the formation of endogenous and exogenous formaldehyde, with a particular focus on the role of formaldehyde in inducing CVD such as arrhythmia, myocardial infarction (MI), heart failure (HF), stroke, AS and diabetic cardiovascular complications. We further discuss the potential pathophysiological mechanism of formaldehyde in CVD. In addition, we highlight the significant role of formaldehyde in heart development by inducing cardiomyocyte apoptosis and oxidative stress. Continued investigation of the unique role of formaldehyde in CVD and heart development is essential for advancing our understanding of CVD and provides a novel therapeutic strategy for the prevention and treatment of CVD and abnormal heart development.

## 2 SOURCES OF FORMALDEHYDE

### 2.1 Endogenous formaldehyde

The continuous generation and degradation of formaldehyde in the body serve to maintain the homeostasis of formaldehyde metabolism. A common mechanism of formaldehyde generation is located in the mitochondria by the action of serine hydroxymethyltransferase 1 and 2.^14^ Serine oxidation by oxidants produced via myeloperoxidase after cardiac ischaemia‐reperfusion (I/R) injury is also a potential source of formaldehyde.^15^ In addition, formaldehyde can be produced by demethylation of DNA and histones catalysed by DNA methyltransferases (DNMTs) and lysine (K) demethylase (KDM).^16^, ^17^ Moreover, fat mass and obesity‐associated (FTO) proteins catalyse the demethylation of 3‐methylamine in single‐stranded DNA to produce formaldehyde as well.^18^

The second endogenous source of formaldehyde is the creatine metabolism pathway, which occurs mainly through the oxidative deamination of creatine to produce SSAO while generating H_2_O_2_ and ammonia.^19^ SSAO represents a collective term for a series of semicarbazide‐sensitive amine oxidases that contain Cu^2+^, which is modified post‐translationally with tyrosine. SSAO exists in soluble form in circulating blood or in membrane‐bound form, mainly in vascular endothelial cells and smooth muscle cells.^20^ SSAO in membrane‐bound form bound within the vascular endothelial cell membrane surface is termed vascular adhesion protein‐1 (VAP‐1), a lymphocyte adhesion receptor that exhibits increased cell surface expression during inflammatory reactions. ^21^ Notably, serum SSAO is maintained at a relatively stable range among healthy adults but is elevated in various pathological states such as congestive HF and types 1 and 2 diabetes mellitus (T1DM, T2DM).^20^

The third source of endogenous formaldehyde is the folate‐mediated one‐carbon metabolism, which is usually related to the participation of methionine in the homocysteine cycle. The physiological significance of this cycle is that N5‐methyltetrahydrofolate, part of the folate‐mediated one‐carbon metabolism that generates formaldehyde, supplies a methyl group to synthesize methionine acid to carry out the methylation reactions that are widespread in the body (Table 1).^22^

**TABLE 1:** The main source of formaldehyde

Source of formaldehyde	Source pathways		
	Intermediate products	Main source	Enzymes/chemical reaction
Endogenous Formaldehyde	DNA and histone	DNMTs^22^ and KDM^23^	Demethylation
	FTO	Single‐stranded DNA^24^	Demethylation of 3‐methylamine
	SSAO	Circulating Blood,^27^ vascular endothelial cells and smooth muscle cells^28^, ^29^ and ect	Oxidative deamination
	Folate‐mediated one‐carbon metabolism	Homocysteine cycle^34^, ^35^	Methionine
Exogenous Formaldehyde	Methanol	Pectin (fresh fruits and vegetables)^49^	Pectin methylsterolase/ fatty acid methyl ester
		Aspartame^50^	Oxidizing reaction
		Burning charcoalmotor /vehicle exhaust/cigarette smoke/antiseptic liquids/plywood adhesives/construction materials^50^, ^51^, ^52^, ^53^	

Abbreviations: DNMTs, DNA methyltransferases; KDM, lysine(K) demethylase (KDM); SSAO, semicarbazide‐sensitive amine oxidase; THF, tetrahydrofolate.

### 2.2 Exogenous formaldehyde

The main source of exogenous formaldehyde in healthy individuals may be through various fruits, vegetables, meat and alcoholic beverages. In 1981, the food additive aspartame was first approved by the United States Food and Drug Administration, gradually becoming the main source of formaldehyde in the diet. Additionally, the pectin present in fresh fruits and vegetables, which functions as a water‐soluble fibre to resist the hydrolysis of gastrointestinal enzymes, is then fermented by the intestinal flora in the large intestine and finally metabolized and absorbed in the human body.^23^ In this process, pectin can be demethylated by pectin methylsterolase to increase the methanol content in the blood and consequently the level of formaldehyde.^24^

In addition, various anthropogenic emissions and artificial products also contain or release formaldehyde. To a certain extent, formaldehyde exists in all aspects of our lives. For example, formaldehyde can be artificially discharged into the air and become a toxic pollutant, such as via burning charcoal, motor vehicle exhaust, cigarette smoke, incineration plants, power plants, or oil refineries (Table 1).^25^ However, when the human body inhales, ingests formaldehyde or absorbs it through the skin, the concentration of formaldehyde in the body will increase, thereby potentially causing substantial damage to important organs in the body.

## 3 FORMALDEHYDE METABOLISM

Formaldehyde in human fluids and tissues maintains the metabolic balance of formaldehyde through the continuous action of formaldehyde‐metabolizing enzymes through multiple metabolic pathways. First, all formaldehyde released by endogenous and exogenous pathways can be combined with tetrahydrofolate (THF) to form 5,10‐methyl THF and then participate in the one‐carbon cycle.^26^ In the cytoplasm, alcohol dehydrogenase (ADH1) uses formaldehyde as a substrate, which is reduced to methanol by NADH or oxidized by formate. Additionally, in the cytoplasm with *ADH5* class III, χ polypeptide (also called formaldehyde dehydrogenase, FDH), encoded by the *ADH5* gene oxidizes S‐hydroxymethyl glutathione to S‐formyl glutathione, which is hydrolysed by S‐formyl glutathione hydrolase, finally generating reduced glutathione and formic acid to participate in metabolism.^27^ Formaldehyde is usually converted into carbon dioxide by taking part in the 1C cycle and is expelled from the lungs or oxidized to formic acid and discharged with urine. Although formaldehyde is rapidly metabolized in the liver and other cells, formaldehyde can also be rapidly produced in different ways in the body as described above to maintain its metabolic steady state.

## 4 FORMALDEHYDE AND CVD

### 4.1 Formaldehyde and arrhythmia

Arrhythmia is caused by abnormal electrical signalling within or exterior to the sinus node (SN), leading to slow signal conduction, blockage, or conduction through abnormal channels, which ultimately results in abnormal heartbeat frequency and rhythm. Studies have found that formaldehyde may have a potential role in causing arrhythmia including sick sinus syndrome (SSS),^8^ atrioventricular block,^27^ ventricular tachycardia, ventricular fibrillation^27^ and atrial fibrillation (AF).^28^ In particular, cardiomyocytes, which comprise excitatory cells that conduct electrical signals and undergo contraction, are extremely sensitive to the toxic effects of formaldehyde. Moreover, exposure to high concentrations of formaldehyde in experimental models can lead to arrhythmia and negative inotropic strength in the heart.^29^

#### 4.1.1 Sick sinus syndrome (SSS)

The precise pathogenesis of SSS, a clinically common refractory arrhythmia, has not yet been clearly elucidated. Clinical and experimental studies have shown that damage to SN cells caused by external factors may elicit cardiac pacing, with conduction dysfunction potentially representing a direct pathological mechanism. In the heart, direct contact of formaldehyde with the SN through various means can cause sinoatrial node dysfunction leading to bradycardia and even cause death from pathological sinus node syndrome. For example, using a cotton swab to directly apply formaldehyde to the SN or a syringe to locally infiltrate the formaldehyde around the sinoatrial node will prolong the sinoatrial node conduction and recovery times. With rapid stimulation of the atria, atrial effective refractory period (ERP) will be further reduced and the duration and induction speed of AF will further increase.^30^, ^31^ The heart rate will be significantly reduced, the wave will disappear and sinus arrest may even occur.

**FIGURE 1:**
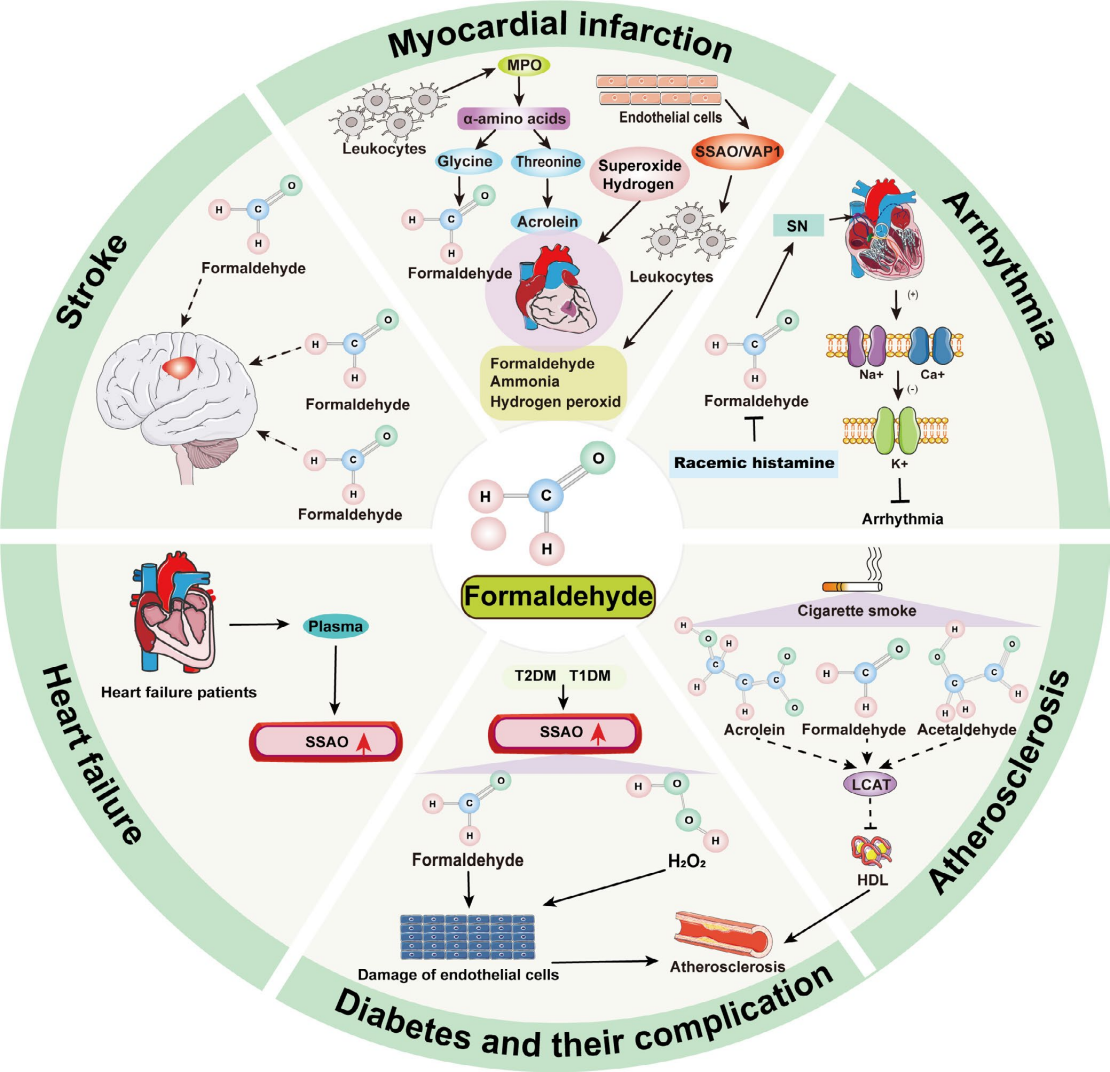
The mechanism of formaldehyde in cardiovascular disease. In the infarcted myocardium, there is MPO released by leukocytes, which can change the oxidation α‐amino produce formaldehyde. For example, MPO‐oxidation products such as glycine (formaldehyde) and threonine (acrolein) exhibit relatively high cytotoxicity. SSAP/VAP1 expressed on the surface of endothelial cells can mediate leukocyte rolling and adhesion in the leukocyte extravasation cascade. These potential mechanisms of action may accelerate the occurrence and development of MI. In the heart, direct contact of formaldehyde with SN or other cardiac conduction systems in different ways may cause SSS or other arrhythmias. Racemic histamine may repolarize the K^+^ current by promoting the influx of Ca^2+^ and Na^+^ to improve arrhythmia caused by formaldehyde. Cigarette smoke contains a large number of reactive aldehydes such as formaldehyde, acetaldehyde and acrolein, which can weaken the reverse transport of cholesterol mediated by HDL by inactivate LCAT and promote the formation of AS. The activity of SSAO in the serum of T1DM patients and T2DM patients is determined by radio‐enzymatic assay. The formaldehyde or H_2_O_2_ generated by SSAO through oxidative deamination can induce or aggravate endothelial cell damage and accelerate the development and severity of diabetic complications such as AS. The formaldehyde present in the air or the formaldehyde produced by SSAO oxidative deamination in the human body may be potentially inducible to stroke. Elevated SSAO activity can be detected in the plasma of patients with congestive heart failure. The higher SSAO activity in patient plasma, the higher the risk of death. HDL, high‐density lipoprotein; LCAT, cholesterol acyltransferase; MPO, myeloperoxidase; SN, sinus node; SSAO, semicarbazide‐sensitive amine oxidase;T1DM, types 1 diabetes mellitus; T2DM, 2 diabetes mellitus

Alternatively, the sinus arrhythmia caused by formaldehyde can be improved by drug intervention. The component racemic higenamine isolated from the traditional Chinese medicine aconite can resolve the SSS induced by formaldehyde, promote blood supply to the SN area, suppress arrhythmia and reduce myocardial ischaemia. Moreover, racemic histamine may repolarize the K^+^ current by promoting the influx of Ca^2+^ and Na^+^ into the action potential platform through shortening the length of the sinus node cycle length (SCL), SN recovery time and total sinoatrial conduction time (TSACT) to accelerate the self‐regulation of sinus node cells and atrioventricular conduction (Figure 1).^28^ In addition, implantation of a tissue‐engineered cardiac pacemaker (TECP) prepared by mixing pacemaker cells derived from cardiac progenitor cells and endothelial progenitor cells into the hearts of rats revealed that TECP could treat sinoatrial node dysfunction induced by formaldehyde through vascularization of the PI3K/Akt‐VEGF/VEGFR signalling pathway.^32^ Furthermore, recent studies have shown that the production of short interfering RNA (siRNA) by lentivirus can inhibit the gene expression of potassium inwardly rectifying channel (*Kir3.1*) in the sinoatrial node area, thereby improving sinus bradycardia caused by formaldehyde‐mediated chemical ablation of the sinoatrial node and increasing the heart rate of mice.^33^

#### 4.1.2 Other types of arrhythmias

In addition to the infiltration of formaldehyde into the SN to induce SSS, infiltration of formaldehyde into other parts of the cardiac conduction system can also cause different degrees of arrhythmia. Formaldehyde injection into the atrioventricular node will not only cause complete atrioventricular block but also lead to spontaneous ventricular arrhythmias.^27^ During this period, after continued stimulation of the right or left stellate ganglion, ventricular tachycardia and even ventricular fibrillation will occur in the epicardium and endocardium.

Inhalation of formaldehyde vapour causes a nasopharyngeal reflex, which stimulates the trigeminal nerve afferent in the nasal mucosa, eliciting a characteristic cardiovascular pattern of vagally mediated bradycardia and sympathetic nerve‐mediated vasoconstriction in the coronary and internal carotid arteries.^34^ Specifically, the severe bradycardia rapidly decreases the heart rate and suppresses the P wave. Ultimately, the QT interval is also prolonged, resulting in non‐sustained polymorphic ventricular tachycardia. Conversely, as triggering of these arrhythmias requires a certain amount of cardio‐neural sympathetic or parasympathetic activity, the application of methylscopolamine can prevent the P‐wave and RT interval changes caused by formaldehyde. In addition, propranolol administration will eliminate ventricular arrhythmia, confirming that this pathology is mediated by sympathetic nerves.^29^, ^34^

Overall, these data indicate that arrhythmias caused by formaldehyde‐induced nasopharyngeal stimulation involve co‐activation of the parasympathetic and sympathetic nerves, increased vagal function in the sinoatrial node and increased sympathetic function in the ventricular myocardium.^35^ In utero, experiments in foetal sheep showed that injection of a certain concentration of formaldehyde into the HIS bundle generated a complete heart block, leading to bradycardia and a sharp drop in cardiac output. Although atrial pacing could restore cardiac output to 80% of that prior to formaldehyde induction, formaldehyde‐induced foetal arrhythmias more readily led to decompensation of heart function, HF and systolic disorders than adult arrhythmias of similar origin.^36^

### 4.2 Myocardial infarction (MI)

MI constitutes a myocardial necrosis caused by acute and persistent coronary ischaemia and hypoxia, which leads to changes in the function of the left ventricle. Inflammation following MI predicts the deterioration of ventricular function and clinical outcome. During the inflammatory response, myeloperoxidase (MPO) is released by activated neutrophils and serves as the main enzyme source for leukocytes to produce oxidants, which are present in the infarcted myocardium. In particular, the oxidation of many common α‐amino acids by the MPO released by leukocytes constitutes an main source of cytotoxic aldehydes in ventricular tissue. MPO‐oxidation products such as glycine (formaldehyde) and threonine (acrolein) exhibit relatively a certain extent cytotoxicity.^37^ Surprisingly, the study found that the oxidation of MPO released by leukocytes had no significant effect on infarction size but increased left ventricular dilation and worsened cardiac function. A series of reactive oxidants (such as formaldehyde) and cytotoxic substances (such as hypochlorous acid) produced by MPO may lead to myocardial dysfunction and poor ventricular remodelling after MI through a variety of potential mechanisms.^15^ Oxidant production after MI was associated with poor clinical outcome. Therefore, the role of oxidants such as superoxide/hydrogen peroxide in MI cannot be ignored.^38^ However, the current study did not compare which oxidant such as superoxide/hydrogen peroxide or the formaldehyde generated by MPO is more harmful to MI remaining further research. SSAO/VAP1 expressed on the surface of endothelial cells and leukocytes, a source of formaldehyde as noted previously, is essential for the role of leukocyte extravasation cascade in mediating leukocyte rolling and adhesion (Figure 1).^20^, ^39^ The use of highly selective inhibitors such as hydralazine (HYD) or 2‐bromoethylamine (2‐BEA) can inhibit the production of SSAO and endogenous formaldehyde, thereby reducing vascular hypoxia damage and reducing the infarct size.^40^, ^41^ This suggests that inhibiting the production of SSAO and endogenous formaldehyde can reduce MI and protect heart function.

Interestingly, studies have reported that formaldehyde is a gender‐specific aldehyde. Compared with male hearts, female hearts have more free formaldehyde at baseline.^42^ It has been proven that the S‐nitrosoglutathione reductase (GSNO‐R) regulated by formaldehyde also has gender differences.^43^ As reported, the female heart has increased GSNO‐R activity, which may be regulated by oestrogen.^44^ GSNO‐R can be used as formaldehyde dehydrogenase to catalyse the oxidation of the spontaneous reaction product (S‐hydroxymethyl glutathione) of formaldehyde. In addition, GSNO‐R also helps to regulate formaldehyde levels by converting formaldehyde to formate, which is then sent to a carbon metabolism cycle and contributes to nucleotide synthesis.^45^ Recent studies further demonstrated that GSNO‐R is a key gender‐dependent mediator for regulating the level of formaldehyde.^46^ GSNO‐R inhibition or gene deletion can reduce the functional recovery of female after I/R injury and increase the infarct size, whereas the male hearts display improved function. Therefore, formaldehyde has been identified as a new active substance in I/R injury, and its different levels in male and female hearts can be regulated by GSNO‐R.

### 4.3 Heart failure (HF)

Myocardial injury caused by MI, cardiomyopathy, haemodynamic overload, inflammation, etc, can result in changes in myocardial structure and function and ultimately lead to reduced cardiac function. SSAO present in human plasma reacts through oxidative deamination to form formaldehyde and H_2_0_2_, and SSAO in the plasma of healthy people can be maintained in a relatively stable range. Elevated SSAO activity can be detected in the plasma of patients with congestive HF and is more substantial in patients with a higher degree of severity.^47^ The increase is even more pronounced in patients with concurrent DM, indicating an additive effect of the two diseases. Through a 5‐year follow‐up study of 372 patients, it was found that the higher the SSAO activity in patient plasma, the higher the risk of death.^48^ Therefore, plasma SSAO could serve as a significant independent indicator of the survival rate following HF, with a risk ratio of 1.5 (Figure 1).

### 4.4 Atherosclerosis (AS)

The initiating factors of AS, a common CVD, include repeated vascular endothelial injury, high cholesterol, oxidative stress, inflammatory factors and oxidized low‐density lipoprotein (OX‐LDL), which damage the vascular endothelium. In addition, studies have shown that endogenous formaldehyde‐induced vascular endothelial injury may also be a potential risk factor for AS. Notably, evaluation of methylamine amination by amine oxidase enzymes in the homogenate of rat aorta and human umbilical artery revealed that SSAO was very active in these two tissues, indicating that methylamine may constitute a potential substrate for SSAO.^49^ Moreover, in rat aorta homogenate and purified porcine aorta SSAO, the use of a specific SSAO inhibitor and high‐performance liquid chromatography to detect methylamine and formaldehyde revealed that SSAO could metabolize methylamine to formaldehyde. Thus, elevated methylamine in the pathological state or increased formaldehyde produced by exogenous sources may lead to increased SSAO activity and excessive production of formaldehyde in tissues.^50^

Formaldehyde is extremely reactive and can induce intramolecular and intermolecular proteins‐protein crosslinks or DNA‐protein crosslinks (DPCs). The cross‐linkage of long‐lasting proteins (such as in the basal membranes of blood vessels) and the production of irreversible adducts can have harmful consequences. For example, SSAO‐mediated formaldehyde generation may be involved in the formation of amyloid and vascular plaques.^7^ In turn, cross‐linking‐mediated vascular sclerosis may constitute a potential risk factor for AS by causing endothelial damage. In mice susceptible to AS, it was found that the increased deamination of methylamine caused a significant increase in formaldehyde production, which further supports that SSAO‐mediated production of this toxin may contribute to AS development.^51^ Furthermore, cigarette smoke contains large numbers of reactive aldehydes such as formaldehyde, acetaldehyde and acrolein. These may inactivate lecithin cholesterol acyltransferase (LCAT) by reacting with the active cysteine‐free sulfhydryl group, which may weaken the reverse transport of cholesterol mediated by high‐density lipoprotein (HDL) and also promote the formation of AS (Figure 1).^52^

#### 4.4.1 Endothelial cells

**FIGURE 2:**
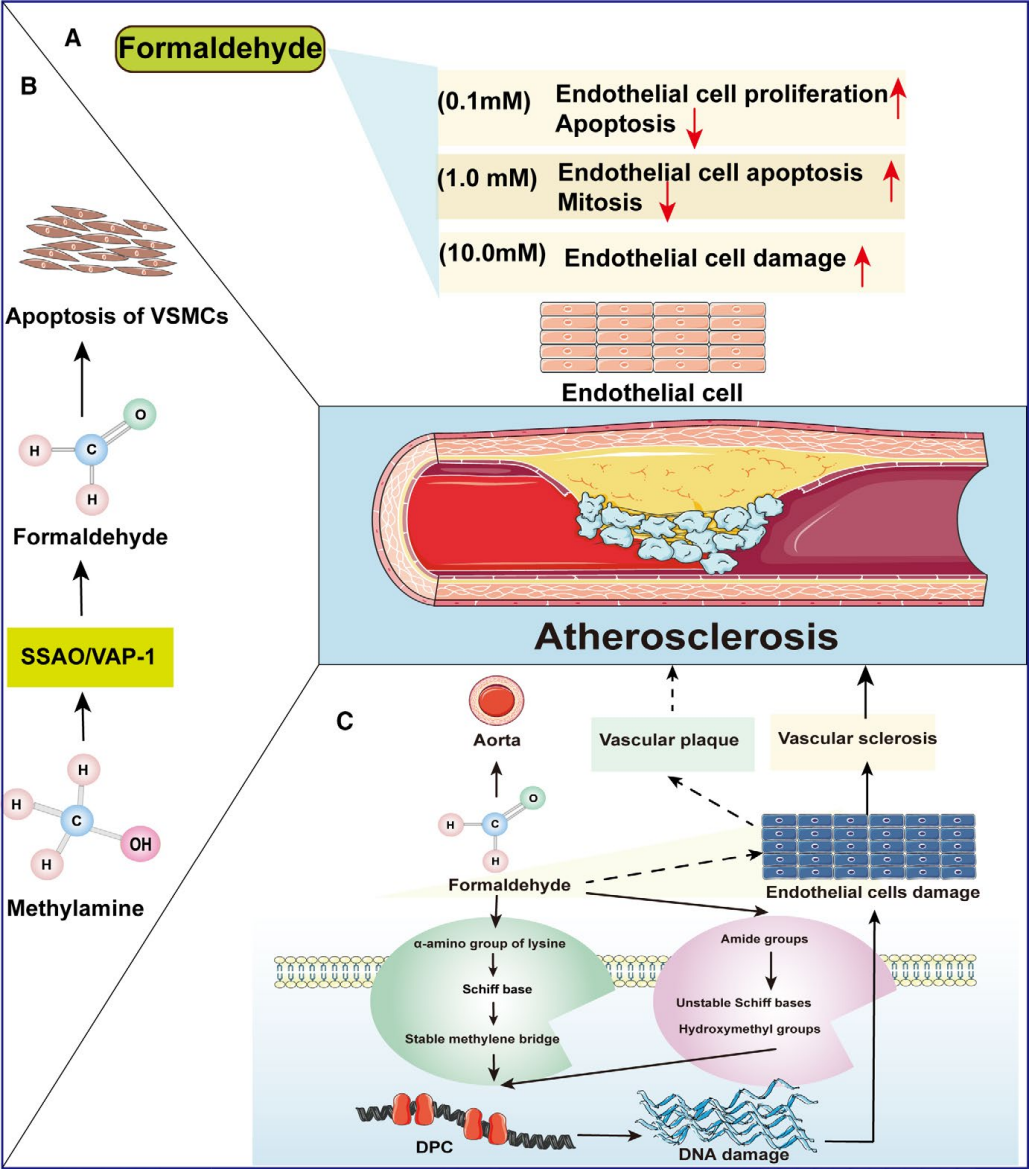
The mechanism of formaldehyde in AS. A, Low‐dose formaldehyde (0.1 mM) can enhance endothelial cell proliferation and reduce apoptotic activity, 1.0 mM formaldehyde can enhance endothelial cell apoptosis and reduce mitosis in a moderate manner. However, high doses of formaldehyde (10.0 mM) can cause strong damage to endothelial cells. Different concentrations of formaldehyde can cause vascular sclerosis and endothelial damage leading to AS. B, SSAO/VAP‐1 mediates the oxidative deamination of methylamine to form formaldehyde and induces VSMC cell apoptosis. SSAO:semicarbazide‐sensitive amine oxidase; VAP‐1,vascular adhesion protein‐1; VSMCs, Vascular Smooth Muscle Cells. C, AG can prevent formaldehyde‐induced B‐amyloid aggregation and eliminate formaldehyde by inhibiting SSAO, thereby reducing the occurrence and development of diabetic vascular complications (such as atherosclerosis). AG, aminoguanidine. D, Formaldehyde can combine with the amino group of lysin in the same or different proteins by interacting with the e‐amino group of lysine to form a Schiff base to further form a stable methylene bridge. Formaldehyde can also interact with amide groups to form unstable Schiff bases and hydroxymethyl groups between crosslinked proteins and DPCs. Moreover, formaldehyde‐induced DPC formation changes the conformation and function of DNA and may also mediate vascular sclerosis, which can damage the endothelium and become a potential risk factor for AS. SSAO‐mediated formaldehyde generation may be involved in the formation of vascular plaques. The continuous accumulation of formaldehyde concentration in the body may also cause damage to endothelial cells and lead to the occurrence of AS

The formaldehyde produced by the action of methylamine and SSAO present in the blood may interact with intravascular components and cause endothelial damage.^51^ Although studies have shown that methylamine is relatively nontoxic in endothelial cells derived from human umbilical veins and bovine pulmonary arteries, the formaldehyde produced by oxidative deamination of methylamine in the presence of SSAO is highly cytotoxic to human endothelial cells and can cause patch damage. Conversely, use of the selective SSAO inhibitor MDL‐72974A could effectively protect endothelial cells from the damage induced by SSAO‐generated methylamine.^53^ In particular, whereas low‐dose formaldehyde (0.1 mM) can enhance endothelial cell proliferation and reduce apoptosis activity, 1.0 mM formaldehyde can enhance endothelial cell apoptosis and reduce mitosis in a moderate manner. However, high doses of formaldehyde (10.0 mM) can cause strong endothelial cell damage^54^ as the generation of DPCs by formaldehyde causes vascular sclerosis and endothelial damage, leading to AS (Figure 2A).

Formaldehyde is a very active chemical substance that can combine with the free amino, amide, or imidazole groups of amino acids in the same or different proteins by interacting with the e‐amino group of lysine to form a Schiff base to further form a stable methylene bridge.^55^ Formaldehyde can also interact with amide groups to form unstable Schiff bases and hydroxymethyl groups between crosslinked proteins and DPCs.^56^ Moreover, formaldehyde‐induced DPC formation changes the conformation and function of DNA. Notably, nucleoproteins cross‐linked with DNA are important for maintaining DNA conformation and are also involved in DNA replication and transcriptional regulation. However, when DPCs appear during the process of DNA replication, the limited repair ability may lead to abnormalities in the expression of important genes, further affecting cell function (Figure 2C).^57^

DPC formation was shown to increase in a concentration‐dependent manner following use of a K^+^/SDS precipitation assay to analyse rat aortic endothelial cells treated with 0.01‐2 mM formaldehyde. In addition, a time‐dependent increase in DPC formation was also observed following treatment with 0.05‐0.1 mM formaldehyde, suggesting that extended exposure to low concentration formaldehyde may contribute to the damage to endothelial cells that occurs during ageing in the body.^57^ In particular, under certain stress conditions, the release of epinephrine will increase; in turn, this will be deaminated by monoamine oxidase to form methylamine. The consequent production of formaldehyde and of irreversible cross‐linking adducts in the tissue may cause damage to the endothelium and subsequent vascular diseases, such as AS.^58^

#### 4.4.2 Vascular smooth muscle cells (VSMCs)

Unlike that expressed by endothelial cells, VAP‐1 expressed on VSMCs does not bind to lymphocytes and is expressed on the plasma membrane of smooth muscle cells.^59^ Notably, injury to vascular endothelial cells may enhance the ability of VSMCs to proliferate and migrate, which may further induce AS formation.^60^ In contrast to endothelial cells that are exposed to SSAO circulating in the serum albeit without endogenous SSAO activity, VSMCs exhibit SSAO activity regardless of serum exposure. Rat VSMCs show SSAO activity and can promote oxidative deamination of methylamine to formaldehyde but are relatively resistant or insensitive to the associated toxic effects, with cytotoxicity observed only at higher methylamine concentrations (LC50 of 0.1 M). However, co‐incubation of soluble SSAO and methylamine in bovine serum has obvious toxic effects on human aortic smooth muscle cells (Figure 2B).^61^

### 4.5 Stroke

Patients with long‐term AS are prone to plaque deposition, which produces arterial thrombosis and triggers stroke. Although SSAO‐mediated deamination of methylamine to formaldehyde in mice susceptible to AS may constitute a potential inducer of AS as described previously,^51^ no statistically significant difference in plasma SSAO activity was observed in 68 patients with stroke and cerebral thrombosis or cerebral infarction compared with that in healthy individuals without stroke.^62^ It is thus possible that changes in the soluble form of SSAO in plasma do not play a major role in causing stroke.

In contrast, long‐term careers in fields in which chemical components such as formaldehyde are used or produced are associated with increased health risks. For example, the furax process is a chemical reaction in which urea and formaldehyde are combined in a catalytic reactor into a urea‐formaldehyde resin polymer under high temperature. Long‐term exposure to formaldehyde or other chemicals may cause stroke. There is a case report of an increased risk of anticardiolipin antibodies and stroke among chemical workers who have been working with urea‐formaldehyde resin for 20 years. Specifically, such exposure may have participated in or caused inflammation, which increases stroke risk (Figure 1).^9^

## 5 FORMALDEHYDE AND THEIR DIABETES COMPLICATIONS

The serum activity of SSAO in the serum of patients with T1DM, T2DM as measured using a radio‐enzymatic assay was significantly increased compared with that of a control group.^63^, ^64^ Moreover, the activity of SSAO in the serum of T2DM with AS was also higher than that of patients with T2DM without AS. Notably, the activity of SSAO in patients with diabetes is related to the risk factors of AS, such as obesity, total cholesterol, high blood pressure and blood sugar.^61^, ^63^ Therefore, the determination of SSAO activity in human serum may constitute a predictive indicator of DM and early AS. In patients with T1DM and T2DM, a significant increase in SSAO activity will lead to increased cytotoxic metabolites such as formaldehyde and H_2_O_2_ to induce or aggravate endothelial cell damage, thereby accelerating AS progression and severity (Figure 1).^48^, ^50^ The up‐regulation of SSAO activity in the serum of T1DM and T2DM may be activated due to the increase of the concentration of its substrate.^65^ Creatine supplementation and combined exercise is the adjuvant treatment for lowering blood sugar in T2DM. However, excessive use of creatine will lead to the production of methylamine, which is deaminated by SSAO to produce cytotoxic compounds such as formaldehyde and H_2_O_2_.^66^ These compounds could lead to the formation of diabetes complications (CVD, stroke, etc).^67^ Interestingly, there are some natural compounds that can reduce the activity of SSAO and the potential adverse effects of creatine metabolism in T2DM.^68^ Caffeine is a natural substance with antioxidant capacity and can act on the imidazoline binding inhibition site of SSAO, thereby inhibiting the activity of SSAO.^69^ Therefore, caffeine intake may balance the activity of SSAO in T2DM supplemented with creatine. Evidence shows that vitamin D could reduce the level of amine oxidase‐monoamine oxidase‐A (MAO‐A),^70^ whereas some MAO inhibitors showed to suppress the activity of SSAO.^70^, ^71^ Therefore, vitamin D may also affect the production of formaldehyde by affecting the activity of SSAO. Although there is no evidence, it can be speculated that the combination of creatine supplementation and vitamin D in the treatment of T2DM seems very promising.

## 6 FORMALDEHYDE AND HEART DEVELOPMENT

As an air pollutant, formaldehyde exerts a toxic effect on the reproduction and development of humans and animals. Inhalation or exposure to formaldehyde prior to conception and during the prenatal or postpartum periods may have a toxic effect on the development of the foetus and cause a series of adverse pregnancy outcome. Exposure to formaldehyde during pregnancy is associated with spontaneous abortion, premature delivery, low birth weight, congenital malformations and other structural abnormalities. However, because it is difficult to directly measure the level of long‐term formaldehyde exposure, research on formaldehyde exposure during pregnancy is limited.

A case‐control study in China found that 118 women diagnosed with miscarriage had significantly higher levels of formaldehyde in their plasma than 191 women who delivered at term.^72^ Notably, the increase in plasma levels of formaldehyde served as a miscarriage‐independent risk factor and the increased formaldehyde content also increased the risk of miscarriage. Formaldehyde can be absorbed through the skin or inhaled or ingested through the mother's respiratory and gastrointestinal tract, respectively, and then transferred to the foetus through the placental circulation. As the clearance of formaldehyde from the foetus is slower than that from the mother, formaldehyde accumulates in the foetal organs.^2^, ^73^

**FIGURE 3:**
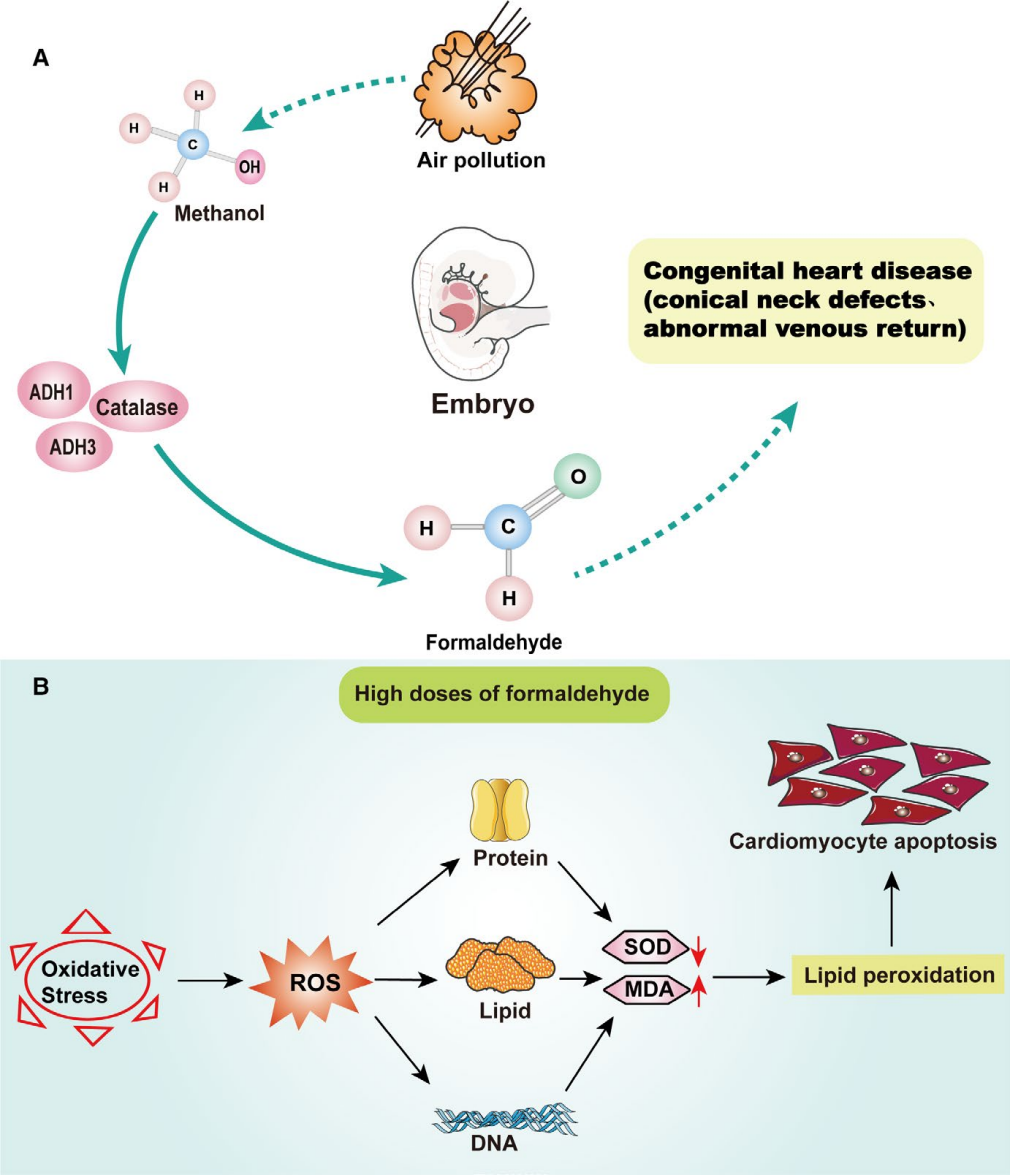
Toxicity mechanism of formaldehyde to embryonic heart development. A, Methanol produced in the air or directly induced embryos with formaldehyde may metabolize methanol to formaldehyde through ADH1, ADH3 and catalase in the embryos, leading to abnormal embryonic heart development such as congenital heart disease. ADH1, alcohol dehydrogenase1; ADH3, formaldehyde dehydrogenase 3. B, Exposure to high doses of formaldehyde during pregnancy may induce oxidative stress in pregnant mice and offspring to produce excessive ROS, which decrease the activity of SOD and increases in MDA, leading to apoptosis of offspring cardiomyocytes. MDA, malondialdehyde; ROS, reactive oxygen species; SOD, superoxide dismutase

In a case‐control study, it was found that exposure to risk factors in the environment may be the cause of the occurrence of cardiovascular malformations in the offspring. Specifically, residential exposure to ambient formaldehyde (>2.42 microg/m^3^) tended to increase the risk of congenital heart malformations by 24% (OR = 1.24; 95% CI 0.81‐2.07).^74^ Notably, pregnant women may spend more time indoors and the amount of gas inhaled or exhaled by pregnant women per minute will thus increase significantly. In addition, a nationwide birth cohort study in Japan showed that the prevalence of congenital heart diseases (CHDs) in pregnant women exposed to house renovation, formaldehyde and organic solvents during pregnancy was 1.1%.^75^ Similarly, a study in China showed that pregnant women undergoing house renovation or moving into newly renovated houses may increase the risk of giving birth to foetuses with certain CHDs.^76^ This suggests that house renovation represents the main form of non‐occupational exposure for this group, which may partly underlie the increase in formaldehyde inhalation.^77^ Maternal exposure to organic dyes, varnishes, or paints also increases the risk of cone septal defects (Figure 3A).^78^

More recently, several reports have clarified possible mechanisms of the occurrence and development of abnormal foetal heart development owing to formaldehyde. Mouse embryos are more sensitive to formaldehyde‐induced cardiac dysplasia than rat embryos. ADH1, ADH3 and catalase can metabolize methanol into formaldehyde and formic acid. The activity of enzymes in mouse embryos during most heart development stages is lower than that in rat embryos, which indicates that the mouse embryos remove formaldehyde at a slower rate (Figure 3A).^79^ Recent studies have found that exposure to high doses of formaldehyde during pregnancy in mice can induce oxidative stress and cardiomyocyte apoptosis in pregnant mice and offspring. However, supplementation with vitamin E during pregnancy can reverse the systemic and cardiomyocyte toxicity induced by formaldehyde. This further reveals that the underlying mechanism of formaldehyde‐induced reproduction and developmental toxicity may occur through DNA, chromatin and cell damage (such as cell death or apoptosis).^80^

Specifically, exposure to formaldehyde can cause oxidative stress in many tissues, leading to the production of reactive oxygen species (ROS). Studies have found that overproduction of ROS or low levels of available antioxidants can result in damage to DNA, proteins and lipids.^81^ This indicates that overproduction of ROS during pregnancy may induce developmental toxicity and lead to various tissue defects in the offspring. Previous research reports indicate that formaldehyde may decrease the activity of superoxide dismutase (SOD) and cause an increase in malondialdehyde (MDA), which is a manifestation of lipid peroxidation.^82^ These results also further suggest that increased oxidative stress induced by formaldehyde exposure can cause cardiomyocyte apoptosis in the offspring, which may be an important mechanism of formaldehyde‐induced cell damage (Figure 3B).

## 7 THE ROLE OF FORMALDEHYDE IN THE DIAGNOSIS AND TREATMENT OF CARDIOVASCULAR DISEASES AND HEART DEVELOPMENT

Formaldehyde may be a potential diagnostic and therapeutic target for CVD and heart development. Plasma SSAO levels are elevated in patients with HF, and the activity of SSAO increases with the progression of disease severity, indicating the endogenous formaldehyde concentration may serve as a biomarker for the diagnosis of HF severity and survival rate.^47^, ^48^ As mentioned above, in the I/R injury model, the different efficacy of drugs to inhibit formaldehyde in male and female may provide a new strategy for clinical treatment with different genders.^42^, ^43^ In addition, an increase in formaldehyde concentration in the air may lead to an increase in CHD,^72^ supporting the correlation between formaldehyde and heart development. Therefore, the detection of formaldehyde concentration in the air and pregnant women's plasma provide a reliable potential indicator for the timely diagnosis of abnormal foetal heart development. Several studies have directly or indirectly proved the toxic effect of different concentration of formaldehyde on CVD. For example, a 90‐minute exposure (formaldehyde:197 ± 79 ppb) in young female who ruled out other diseases found that short‐term exposure to formaldehyde would adversely affect vascular function.^83^ A national birth cohort study in Japan showed that during pregnancy exposed to house decoration, formaldehyde and organic solvents, the probability of the offspring suffering from CHDs is 1.1%.^75^ Additionally, in normal and hypertrophic heart rats, intravenous infusion of 3.7% formaldehyde solution at 10 µl (3.7 mg)/kg/min was found to cause different degrees of acute HF, especially in rats with hypertrophic heart.^84^ The risk of CHDs for residents exposed to environmental formaldehyde (>2.42 microg/m^3^) rised by 24% (OR = 1.24;95% CI 0.81‐2.07).^74^ These studies imply the toxic effect of certain amount of formaldehyde on CVD and heart development. However, when hyperlipidaemia, obesity, diabetes, hypertension and other pressures exist, people are unable to determine whether formaldehyde is the main factor leading to CVD or heart development. Therefore, the molecular mechanisms and physiological significance of formaldehyde in the CVD and heart development are still needed for more investigation.

## 8 CONCLUSION AND FUTURE PERSPECTIVES

As an air pollutant that affects human health, formaldehyde widely exists in the environment. CVD is one of the causes of death and disability that endangers human health worldwide. The role of formaldehyde, especially endogenous formaldehyde in CVD has been extensively studied. Endogenous formaldehyde can interact with nucleic acids and proteins to form an irreversible cross‐linked complex, which affects the structure and function of proteins and genes, leading to vascular, pathological and myocardial damage to participate in the occurrence of CVD. Abnormal heart development caused by formaldehyde exposure may also pose a threat to the health of human offspring. The toxic effects of formaldehyde exposure on the heart development of offspring have been studied in humans and animals. Numerous studies have focused on the effects of formaldehyde exposure in the environment on the growth and development of pregnant women and their foetuses, albeit with relatively few studies on heart development. Long‐term exposure of pregnant women to relatively high levels of formaldehyde can cause foetal cardiac dysplasia, such as conical neck defects and abnormal venous return. Animal‐related experimental studies have shown that formaldehyde exposure is strongly linked to heart development. Formaldehyde exposure in pregnant mice induced oxidative stress and apoptosis of cardiomyocytes in offspring. Moreover, ROS may cause changes in functions such as cardiomyocyte apoptosis,^85^ and pyropyosis.^86^ However, whether these changes are involved in formaldehyde‐induced abnormal heart development has not been confirmed. Further animal and mechanistic studies are still needed to fill the gaps related to the effects of formaldehyde exposure in heart development research.

Moreover, emerging evidence demonstrated that formaldehyde also participates in the regulation of toxicogenomics and proteomics. It can mediate DNA and RNA damage and regulation of gene differential expression. For example, basic helix‐loop‐helix domain‐containing protein class B2 (BHLHB2), CyclinL1 (CCNL1), cutaneous T‐cell lymphoma‐associated tumour antigen (SE20‐4), tribbles pseudokinase 1 (also known as C8FW), polo‐like kinase‐2 (PLK2) and serum‐and glucocorticoid‐induced protein kinase 1 (SGK) were upregulated in human tracheal fibroblasts exposed to formaldehyde.^87^ These genes respond to the differential expression of formaldehyde and may be potential biomarkers of formaldehyde exposure. In addition, proteomic studies have found that formaldehyde has epigenetic effects leading to histone H3 phosphorylation.^88^ However, there are no toxicogenomics and proteomics studies on formaldehyde in CVD and heart development, which need further deep investigation.

Formaldehyde has long been known as a toxin and carcinogen, but its beneficial effects have recently been recognized. This is mainly due to the lack of direct evidence to support a beneficial effect of formaldehyde on specific biomolecules or cellular mechanisms rather than a non‐specific destructive effect. The endogenous availability of formaldehyde indicates its potential physiological effects. However, because of the methodological complexity associated to real‐time, patio‐temporal measurements of formaldehyde in living cells and tissues, these effects have been barely deciphered. Accordingly, Rongfeng Zhu developed a genetically encoded, reaction‐based formaldehyde sensor‐FAsor, which makes it possible to display formaldehyde in different living species, including mammalian cells and mouse brain tissue. In addition, they found that formaldehyde induces the transcriptional activation of HxlR through helical cross‐linking reaction through FAsor.^89^ This investigation will facilitate the research progress of the significant involvement of formaldehyde in diseases.

In recent years, the roles of non‐coding RNAs (ncRNAs), including microRNAs, long non‐coding (lnc) RNAs and circulating (circ) RNAs, in disease pathogenesis, regulatory mechanisms and therapeutic applications have gradually become hot areas of research.^90^, ^91^, ^92^, ^93^ Moreover, ncRNA has been shown to have an important regulatory role in CVD,^92^, ^94^, ^95^, ^96^, ^97^, ^98^, ^99^, ^100^, ^101^, ^102^, ^103^, ^104^, ^105^ tumours,^106^ inflammation,^103^, ^107^ and heart development.^108^, ^109^ Increasing studies have shown that ncRNAs constitute potentially important players in biological regulation.^110^ In turn, studies have found that formaldehyde can also participate in the occurrence and development of diseases by regulating ncRNA. MicroRNA is the most widely studied ncRNA that can affect various diseases caused by environmental exposure. For example, inhalation exposure to formaldehyde in the nasal epithelium of nonhuman primates can significantly disrupt miRNA expression profiles. Prediction and analysis of the transcriptional targets of the miRNA with the greatest increase (miR‐125b) and decrease (miR‐142‐3p) in expression revealed that these may affect the signal transduction of apoptosis.^111^ Moreover, genome‐wide miRNA expression profiles were evaluated in nasal respiratory epithelium, circulating white blood cells (WBCs) and bone marrow (BM) exposed to formaldehyde inhalation. It was found that the inflammatory signal induced by formaldehyde originated from the nose, which may further drive the WBC effect.^112^ Furthermore, studying the expression profile of miRNAs exposed to gaseous formaldehyde in human lung cells revealed that 89 miRNAs were significantly downregulated and that the alterations of these miRNAs may be involved in the signal transduction pathways related to cancer, inflammatory response and endocrine system regulation.^113^ These findings indicate that formaldehyde exposure can change the expression profile of miRNAs in different cells or tissues, which may serve as a potential pathogenesis of formaldehyde‐induced different diseases. Such results also indicate that formaldehyde may facilitate the occurrence and progression of CVD and heart development by regulating the expression of ncRNA. However, research on ncRNA related to formaldehyde in CVD and heart development remains insufficient.

Therefore, it is critical that further research be performed with regard to formaldehyde and ncRNA in combination to explore the potential molecular mechanisms in CVD and heart development. Continued investigation of the unique role of formaldehyde in CVD and heart development is essential for advancing our understanding of CVD and is expected to provide novel therapeutic strategies for the treatment and prevention of CVD and heart development.

## CONFLICT OF INTEREST

The authors have declared that no competing interest exists.

## AUTHOR CONTRIBUTION

**Ying Zhang:** Data curation (lead); Formal analysis (lead); Investigation (lead); Methodology (lead); Resources (lead); Writing‐original draft (lead). **Yanyan Yang:** Conceptualization (equal); Funding acquisition (equal); Writing‐review & editing (equal). **Xiangqin he:** Methodology (supporting); Resources (supporting); Software (supporting). **Panyu Yang:** Formal analysis (supporting); Investigation (supporting); Methodology (supporting); Resources (supporting). **Tingyu Zong:** Resources (supporting); Software (supporting). **Pin sun:** Visualization (supporting). **Rui‐Cong Sun:** Resources (supporting). **Tao Yu:** Conceptualization (lead); Funding acquisition (equal); Project administration (equal); Supervision (equal); Validation (equal); Visualization (equal); Writing‐review & editing (lead). **Zhirong jiang:** Funding acquisition (equal); Project administration (equal); Supervision (equal); Visualization (equal).

## Data Availability

Data sharing not applicable to this article as no data sets were generated or analysed during the current study.
